# Endophytic bacterial community dynamics in sweet cherry in vitro shoot culture and their role in shoot adaptation after cryopreservation

**DOI:** 10.1186/s12870-024-05866-z

**Published:** 2024-11-29

**Authors:** Jurgita Vinskienė, Inga Tamošiūnė, Rytis Rugienius, Elena Andriūnaitė, Vidmantas Stanys, Danas Baniulis

**Affiliations:** Institute of Horticulture, Lithuanian Research Centre for Agriculture and Forestry, Kaunas str. 30, Babtai Kaunas reg., 54333 Lithuania

**Keywords:** Cold hardening, Endophytic bacteria, Germplasm, Meristem, Metataxonomic analysis, Regrowth

## Abstract

**Background:**

In vitro cultivation and cryopreservation techniques are essential tools for genetic diversity conservation and pathogen-free plant propagation of horticultural crops. The optimisation of cryopreservation protocols typically focuses on minimising the negative effects of pretreatment with cryoprotectors (CPs), cryogenic freezing (CF) treatment, and recovery procedures on explants. However, the impact of in vitro and CF techniques on plant-associated microbiota remains poorly understood, and their potential to improve plant adaptation after cryopreservation is underexplored. The aim of the present study was to investigate in vitro shoot culture and cryopreservation-induced changes in the endophytic bacterial diversity of two sweet cherry cultivars and to assess the potential of an inoculum of bacterial isolates to improve the growth of shoot culture after CF.

**Results:**

Cultivars ‘Sunburst’ and ‘Mindaugė’ showed different responses to cold hardening preconditioning as well as different survival and regrowth rates after cryopreservation. Metataxonomic analysis revealed variation in the abundance and taxonomic composition of bacteria assigned to 35 families in samples of field-grown tree leaves, dormant buds, and in vitro shoot culture before and after CF treatment. *Bacillaceae* and *Enterobacteriaceae* bacteria were predominant in the leaf samples of both cultivars. For ‘Sunburst’, *Pseudomonadaceae* and *Sphingomonadaceae* bacteria were dominant in dormant buds and in vitro shoots, respectively, while *Burkholderiaceae* was largely predominant in the shoots following CF treatment. Conversely, ‘Mindaugė’ tissues exhibited more consistent colonisation by *Bacillaceae* and *Enterobacteriaceae* across the experimental groups, except for in vitro shoots where *Mycobacteriaceae* prevailed. A pure bacterial isolate inoculum was applied to the ‘Mindaugė’ shoot culture to counter the CF treatment-induced suppression of shoot growth (~ 40%). Cocultivation with *Brevibacterium* sp. S1-2, *Bacillus cereus* S1-3, or *B. toyonensis* Nt18 increased the shoot leaf area from 48 to 75%.

**Conclusions:**

This study revealed that endophytic bacterial diversity is significantly reduced under in vitro conditions, often leading to a genotype-specific increase in the abundance and dominance of bacteria attributed to a single bacterial family. Moreover, shoot cocultivation with endophytic bacterial isolates has potential for improving the recovery of in vitro shoots after cryopreservation.

**Supplementary Information:**

The online version contains supplementary material available at 10.1186/s12870-024-05866-z.

## Background

Sweet cherry (*Prunus avium* L.) is an economically important fruit tree species of the Rosaceae family that bears nutritious stone fruits rich in vitamins, carotenoids, and polyphenol compounds [1]. Although most of the cherries on the market are derived from only a few dozen varieties, a large diversity of cherry landraces is found in Europe [2], which, considering the impending climate change, could represent a valuable resource of alleles for the breeding of traits such as tolerance to biotic or abiotic factors. Cryogenic freezing (CF) is the only ex situ conservation method for the long-term preservation of the biodiversity of clonal crops [3]; therefore, cryopreservation is an essential technique for conservation of the diversity of traditional sweet cherry varieties. In addition, in vitro shoot tip culture and cryopreservation are reliable and affordable methods for maintaining virus and graft-transmissible pathogen-free nuclear stocks used for the preparation of certified plant propagation material for vegetatively propagated crops.

Vitrification-based cryopreservation is commonly used for shoot tips of clonally propagated horticultural plants, and a variety of protocols have been described [4–10]. Several protocols for vitrification-based cryopreservation of shoot tips of sweet cherry or related *Prunus* species have been developed previously, including preconditioning of donor plants or shoot tips through cold hardening, exposure to high sugar levels, or anti-oxidants required to improve resilience to stresses inflicted by the cryopreservation procedure [11–13]. Although satisfactory results could be achieved without cold hardening for certain genotypes [11, 12, 14–17], most *Prunus* cryopreservation protocols use in vitro shoot cold hardening for several weeks before excision of shoot tips. Furthermore, the preculture of shoot tips on solid media supplemented with a high level of sucrose or in combination with other cryoprotectants is essential for the efficient survival and regrowth of sweet cherry [12, 18], *P. jamasakura* [19], and interspecific rootstocks of *Prunus* sp. [11, 20], as well as related species of the genus such as the plum species *P. domestica* [16, 17, 21], *P. cerasifera* [14, 17], *P. insititia* [17], and almond *P. dulcis* [22–24].

While the main focus of cryopreservation protocol optimisation has often been the minimisation of the negative effect of cryoprotectant pretreatment steps, freezing, and recovery procedures, the growth vigour and proliferation capacity of regenerated shoots have rarely been assessed. Nonetheless, enduring effects of cryopreservation on the growth and multiplication parameters of regenerated shoots of *Prunus* [14, 16, 25–28] as well as other plant species [29–32] have been reported. These changes are usually attributed either to the residual physiological response to stress induced by cryoprotectants, freezing procedures, or the changed sanitary status of the in vitro culture [13, 33]. Indeed, contamination by microbial agents and pathogens or the presence of bacteria of a putative endophytic origin in plant in vitro tissue culture is relatively common, especially for cultures initiated from surface-sterilised explants or seeds without systemic antimicrobial treatment using antibiotics [34–38]. Additionally, a negative effect of bacterial endophytes on tissue recovery after cryopreservation has been reported [39, 40]. However, the presence of bacteria in plant in vitro culture presents a two-sided argument and could be designated either as microbial infection when it leads to the development of disease symptoms or endophytic colonisation when there is no adverse effect on the plant host.

Bacteria that constitute the plant microbiome are known to play an important role in plant adaptation and survival under unfavourable conditions [41], and it has been reported that complementation of axenic culture with endophytic bacteria could mitigate stress, improve growth or rooting, and facilitate acclimation under in vitro conditions [36, 42–46]. Quambusch et al. [47] described differences in endophytic bacteria species composition associated with the propagation properties of sweet cherry in in vitro shoot culture. Additionally, stimulation of the rooting of sweet cherry in vitro by endophytic bacteria inoculum has been reported [47, 48]. However, there is a lack of data about the effect of in vitro cultivation and cryopreservation procedures on plant endophytic microbiota composition, and the potential of inoculating plant growth-promoting bacteria for plant recovery and adaptation after cryopreservation has not been directly addressed.

The aim of the present study was to investigate in vitro shoot culture and cryopreservation-induced changes in the endophytic bacterial diversity of two sweet cherry tissues and to assess the potential of an inoculum of bacterial isolates to improve the growth of shoot culture after cryopreservation. Two partially unrelated sweet cherry genotypes, cultivars (cvs.) ‘Sunburst’ and ‘Mindaugė’, which showed distinct responses to cold-hardening and CF treatment, were used in the present study. Endophytic bacterial diversity in in vitro shoot culture before and after cryopreservation procedures was compared to that in the leaves and buds of field-grown plants using a metataxonomic approach. Furthermore, the potential application of the inoculum of pure bacterial isolates to improve the growth of shoot culture after CF was investigated.

## Materials and methods

### Plant material and in vitro shoot culture

Sweet cherry (*Prunus avium* L.) cvs. Mindaugė and Sunburst were obtained from the collection of genetic resources of the Lithuanian Research Centre for Agriculture and Forestry. For experiments bud and leaf samples were collected from field-grown trees. To establish in vitro shoot culture, bud explants were rinsed under running tap water and surface sterilised by submerging them in mercuric chloride solution for 10 min, followed by washing with sterile deionised water. Sterilised explants were cultured using sweet cherry in vitro shoot propagation conditions: basal Murashige and Skoog (MS) [49] media supplemented with the vitamins thiamine, pyridoxine, and nicotinic acid at 0.5 mg l^− 1^ each, 0.75 mg l^− 1^ benzylaminopurine, 1 mg l^− 1^ ascorbic acid, 3% sucrose, and 0.7% plant agar (pH 5.8) at 22 ± 3 °C under illumination at 60–70 µmol m^− 2^ s^− 1^ and a 16-hour photoperiod. The established shoot culture was transferred to fresh media every four weeks.

### Cryogenic freezing of in vitro shoot tips

The effect of cold hardening preconditioning on the resilience of sweet cherry shoots to cryopreservation treatment was assessed. For cold hardening, in vitro shoots were grown for two weeks under in vitro shoot propagation conditions and then transferred to 4 ± 1 °C and a short-day (8-hour) photoperiod of 15–20 µmol m^− 2^ s^− 1^ for two weeks. Unhardened shoots were grown under in vitro shoot propagation conditions for 2 weeks before the cryopreservation experiment.

Sample pretreatment with cryoprotectants (CPs) and loading solution was performed using a modified vitrification technique [12, 18, 50]. Apical shoot tips (2–3 mm in length) were excised from the shoots and plated onto preculture medium (MS medium supplemented with 0.4 M sucrose, 2 M glycerol, and 1% plant agar) for 2 days at 22 °C in the dark. Shoot tips from the preculture medium were transferred to cryo-tubes (10 shoot tips per tube) filled with 0.9 ml of loading solution (MS medium supplemented with 0.8 M sucrose and 2 M glycerol) and incubated for 30 min at room temperature. The loading solution was replaced with 0.9 ml of plant vitrification solution (PVS2) [51] (MS medium, 4.1 M glycerol, 2.7 M ethylene glycol, 2.2 M dimethyl sulfoxide, and 0.64 M sucrose), and the tubes were kept on ice for 40 min.

Tubes with CP-treated shoot tips were placed in a Styrofoam box and subjected to slow freezing to -40 °C at a rate of 0.2–0.3 °C/min in a freezer before transferring the tubes to liquid nitrogen for half an hour. The samples were thawed in a 40 °C water bath for 2 min and rinsed with liquid MS medium containing 1 M sucrose for 20 min. To assess the effect of CP treatment on the survival and regrowth of shoot tips, freezing and thawing steps were omitted, and the samples were rinsed with sucrose solution immediately after incubation with PVS2.

After the excess sucrose solution was removed by placing the shoot tips on sterile filter paper, the shoot tips were transferred to a Petri dish with MS medium supplemented with 1 g l^− 1^ polyvinylpyrrolidone and 1% plant agar and incubated at 22 °C in the dark for 1 week, followed by incubation under 60–70 µmol m^− 2^ s^− 1^ illumination and a 16-hour photoperiod for 2 weeks. The survival rate of apical shoot tips was estimated based on the tissue colour of the shoot tips (green and brown corresponding to viable and necrotic shoot tips, respectively). Shoot tips were transferred to shoot propagation medium and maintained under in vitro shoot propagation conditions. The shoot regrowth rate was estimated after 4 weeks. Shoot tips that gave rise to whole in vitro plantlets and regenerated were considered recovered.

The leaf area of shoots grown under in vitro shoot propagation conditions (control) and recovered after CF treatment (CF-treated) was evaluated. Shoot images were collected every two or three days for three weeks in total, and leaf area was estimated using ImageJ software [52]. Shoot fresh weight (mg) and the number of shoots per explant were measured at the end of the experiment.

### Tissue oxidative injury analysis

Oxidative injury of shoot cellular membranes was estimated based on quantitative analysis of the accumulation of the lipid peroxidation product malondialdehyde (MDA), according to Hodges et al. [53] and Jagendorf and Takabe [54]. Briefly, homogenised frozen shoot powder was extracted with 50 mM Tris-HCl, pH 7.4, containing 1.5% polyvinylpolypyrrolidone for 30 min at 4 °C and centrifuged at 10,000 × g for 15 min at 4 °C. Equal amounts of tissue extract and 0.5% thiobarbituric acid in 20% trichloroacetic acid were mixed, heated at 95 °C for 30 min, cooled on ice, and centrifuged at 10,000 × g for 5 min. The absorbance measured at 532 nm was corrected by subtracting the absorbance value at 600 nm, and the MDA concentration was estimated using e = 155 mM^− 1^ cm^− 1^. The absence of interference from the absorbance of the anthocyanins at 532 nm was verified using control samples without thiobarbituric acid.

### In vitro cocultivation with endophytic bacteria

After cryopreservation, the shoot culture of cv. Mindaugė was maintained under in vitro shoot propagation conditions as described above. Four-week-old shoots were inoculated with growth-promoting bacterial isolates of *Bacillus toyonensis* Nt18 [43], *Brevibacterium* sp. S1-2, *Bacillus cereus* S1-3, and *Pseudomonas* sp. L1-2 (unpublished data). Shoot inoculation experiments with some modifications were carried out as described previously by Tamošiūnė et al. [46]. Briefly, the bacterial inoculum was grown in lysogeny broth (LB) [55] at 25 °C to the exponential growth phase. Bacteria were sedimented via centrifugation and resuspended in MS media at a concentration of ~ 10^7^ colony-forming units (CFUs) mL^− 1^. The top part of the shoot (∼1 cm) was cut and immersed in a bacterial suspension for 4–5 min. MS medium was used for the control without bacterial inoculation. The shoots were transferred to fresh shoot cultivation medium and maintained under in vitro shoot propagation conditions, and shoot fresh weight and leaf area were assessed after 12 days of cocultivation.

### Sample preparation for microbiome analysis

For microbiome analysis, bud samples were collected at the end of March, and leaf samples were collected in early June and at the end of August 2021. The leaf sample surface was sterilised using a modified protocol designed by Mendes et al. [56]. The leaves were washed with running tap water to remove epiphytic microorganisms and contamination. The samples were sequentially washed by shaking in 75% ethanol for 5 min, rinsed with sterile distilled water, and then submerged in 4% sodium hypochlorite solution for 5 min. Sterilised leaves were rinsed with sterile distilled water again and 75% ethanol for 30 s. The buds were sterilised as described by Hata et al. [57]. Briefly, buds were incubated in 70% ethanol for 5 min, 15% hydrogen peroxide for 20 min, and 70% ethanol for 1 min and then washed with sterile distilled water five times. Then, the upper layer of the scales was peeled off mechanically with a scalpel. To confirm sterility, 100 µl of the last rinsed water was inoculated on LB plates and incubated at room temperature for three days. Sterilisation was considered successful when no colonies were observed. Sterilised leaves and buds were stored at -70 °C.

Samples of shoots grown under in vitro shoot propagation conditions and shoots recovered after cryogenic freezing were collected during the active growth stage, approximately 2–3 weeks after transfer to fresh medium.

### DNA extraction and bacterial 16 S rRNA-based microbiome analysis

Samples for DNA extraction were flash-frozen in liquid N_2_, ground to a fine powder, and stored at -70 °C. Bacterial DNA was extracted using the method described by Ding et al. [58], except 0.2 g of starting material was used, and the final elution was performed in 20 µl of nuclease-free water.

Variable domain 4 of the *16 S rRNA* gene is often used for bacterial diversity analysis due to its effective taxonomic coverage [45]. A fragment of this gene was amplified using specific primers (V4F CCAGCAGCCGCGGTAATA and V4R GGACTACCAGGGTATCTAATCCTGT) under the following cycling conditions: 25 cycles of 30 s of denaturation at 95 °C, 30 s of annealing at 58 °C and 20 s of extension at 72 °C. Negative (no DNA template) and positive (*E. coli* DNA*)* controls were used to confirm the specificity of amplification.

The DNA library preparation procedure was based on the 16 S Metagenomic Sequencing Library Preparation Protocol (Thermo Fisher Scientific, USA) and performed as described previously [45]. An equal volume of each sample adjusted to 10 pM was combined, and emulsion PCR was carried out using Ion OneTouch 2 System and Ion PGM Hi-Q View OT2 Kit (Thermo-Fisher Scientific, USA). The amplified clonal libraries were enriched using Ion PGM Enrichment Beads on Ion OneTouch ES instrument (Thermo Fisher Scientific, USA). Sequencing was performed on the Ion Personal Genome Machine system using Ion 318 v.2 chip and Ion PGM Hi-Q Sequencing Kit (Thermo Fisher Scientific, USA).

Base calling and run demultiplexing were performed by Torrent Suite v.5.15 (Thermo Fisher Scientific, USA) with default parameters. Sequencing data were processed using the 16 S metagenomic workflow of Ion Reporter Software v.5.20.2.0 (Thermo Fisher Scientific, USA). The reads were trimmed by primers at both ends. The threshold for unique reads was set to 10. Taxonomic identification was performed using MicroSEQ 16 S Reference Library v.2013.1 and Greengenes v.13.5 databases. The threshold value for percentage identity for genus and species ID was 97%.

### Data analysis

For cryogenic freezing experiments, 2–4 replicates of 10 shoot tips were used for each experimental group, and the experiments were repeated at least twice to confirm reproducibility. For growth and stress parameter analysis, 4 replicates of 12 shoots were used for each experimental group. The mean and the standard error of the mean (SEM) were estimated for survival, recovery, growth, and oxidative injury parameters, and the statistically significant difference (*p* < 0.05) between the means was assessed by one-way analysis of variance (ANOVA) and a Tukey post-hoc test (Prism, GraphPad Software Ltd.).

For 16 S rRNA-based microbiome analysis, 3 biological replicates were used for each experimental group. Statistical data analysis was performed using the Microbiome Analyst server [59]. The data were rarefied to the minimum library size, and total sum normalisation was applied to the taxonomic count data by dividing the feature read counts by the total number of reads in each sample. Nonmetric multidimensional scaling (NMDS) was used to compare the diversity of bacterial communities among the experimental groups.

## Results

### Survival and regrowth of apical shoot tips after cryogenic freezing treatment

**Fig. 1:**
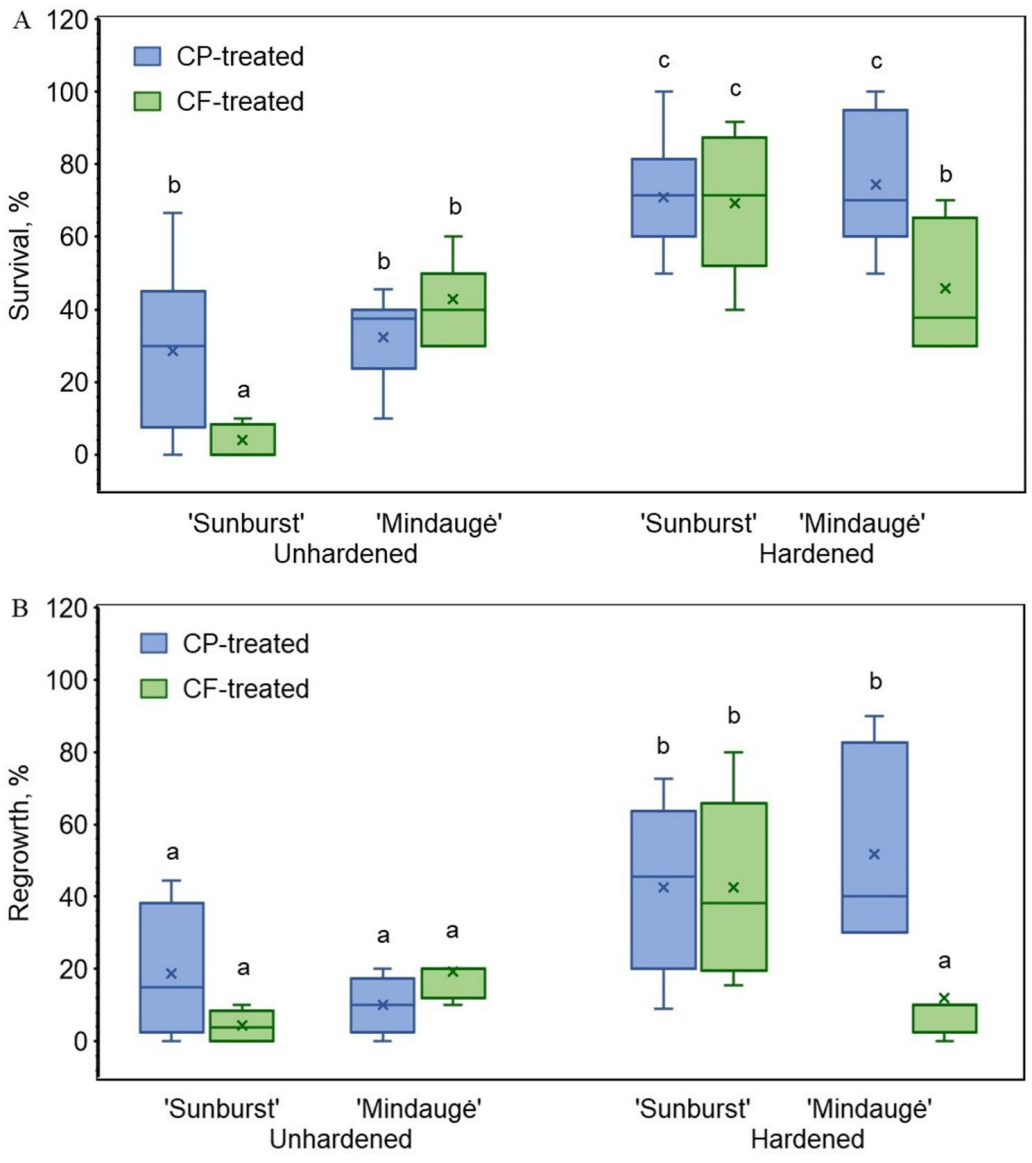
The survival (**A**) and regrowth (**B**) rates of apical shoot tips after cryopreservation. Apical shoot tips of sweet cherry cvs. Sunburst and Mindaugė were pretreated with cryoprotectant solution (CP-treated) and cryogenic freezing (CF-treated) with or without cold hardening preconditioning. The data are shown as boxplots representing the means, medians, minimum and maximum scores, and lower and upper quartiles; different letters denote significant differences between the analysed groups (*p* ≤ 0.05)

The apical shoot tips were prepared from cold unhardened shoots of sweet cherry. Cvs. Sunburst and Mindaugė showed similar responses to the cryopreservation pretreatment procedure, and the survival rates were 4% and 43%, while the regrowth rates were 4% and 19%, respectively (Fig. 1; electronic supplementary material, Table S1).

Considering the low survival and regrowth rates observed for the unhardened shoots, preconditioning treatment at 4 °C for two weeks before apical shoot tip excision was used to improve tolerance to CP and cryopreservation treatment. A significant increase (2–3 fold) in the initial survival rate and regrowth of the apical shoot tips was observed for the cold-hardened shoots of both genotypes after CP pretreatment (Fig. 1). However, the response to CF treatment improved only for cv. Sunburst (69% survival and 43% regrowth), and cold hardening did not result in any significant improvement in the shoot tips of cv. Mindaugė (46% survival and 12% regrowth).

### Effect of cryogenic freezing treatment on shoot growth and tissue stress injury

**Fig. 2:**
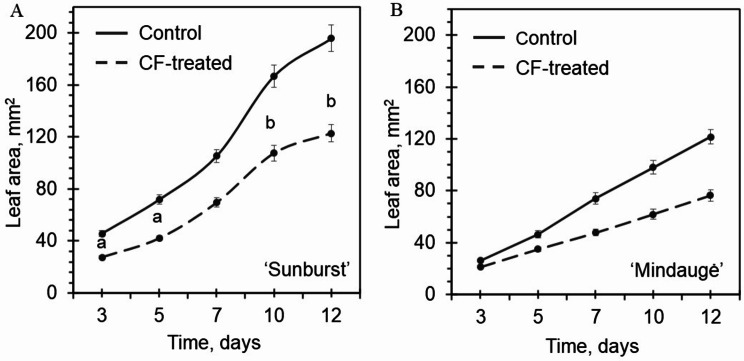
Cryogenic freezing effect on in vitro shoot growth. Leaf area increase of control shoots and shoots regenerated from CF-treated apical shoot tips of sweet cherry cvs. Sunburst (**A**) and Mindaugė (**B**) plants were cultivated for 12 days after transfer to fresh medium. The data are presented as the mean ± SEM; the experimental groups at all time points differed significantly (*p* ≤ 0.05); the same letter indicates no significant difference among the time points (*p* ≤ 0.05)

During 12 days of cultivation, the leaf area and rate of increase in the leaf area of cv. Sunburst control shoots (grown under in vitro shoot propagation conditions) were 1.5–1.6-fold greater than those of cv. Mindaugė (Fig. 2). Conversely, the shoot propagation index of cv. Mindaugė control shoots was 3.3-fold greater than that of cv. Sunburst (electronic supplementary material, Fig. S3B). For shoots that recovered after CF treatment, the leaf area increase was reduced by ~ 35% and ~ 46% for cv. Sunburst and cv. Mindaugė, respectively (Fig. 2A). Although CF treatment reduced the proliferation rate 2.5-fold for cv. Mindaugė, it had a significant effect on stimulating shoot proliferation for cv. Sunburst, resulting in a 2-fold increase in the number of shoots per explant (electronic supplementary material, Fig. S3B).

**Fig. 3:**
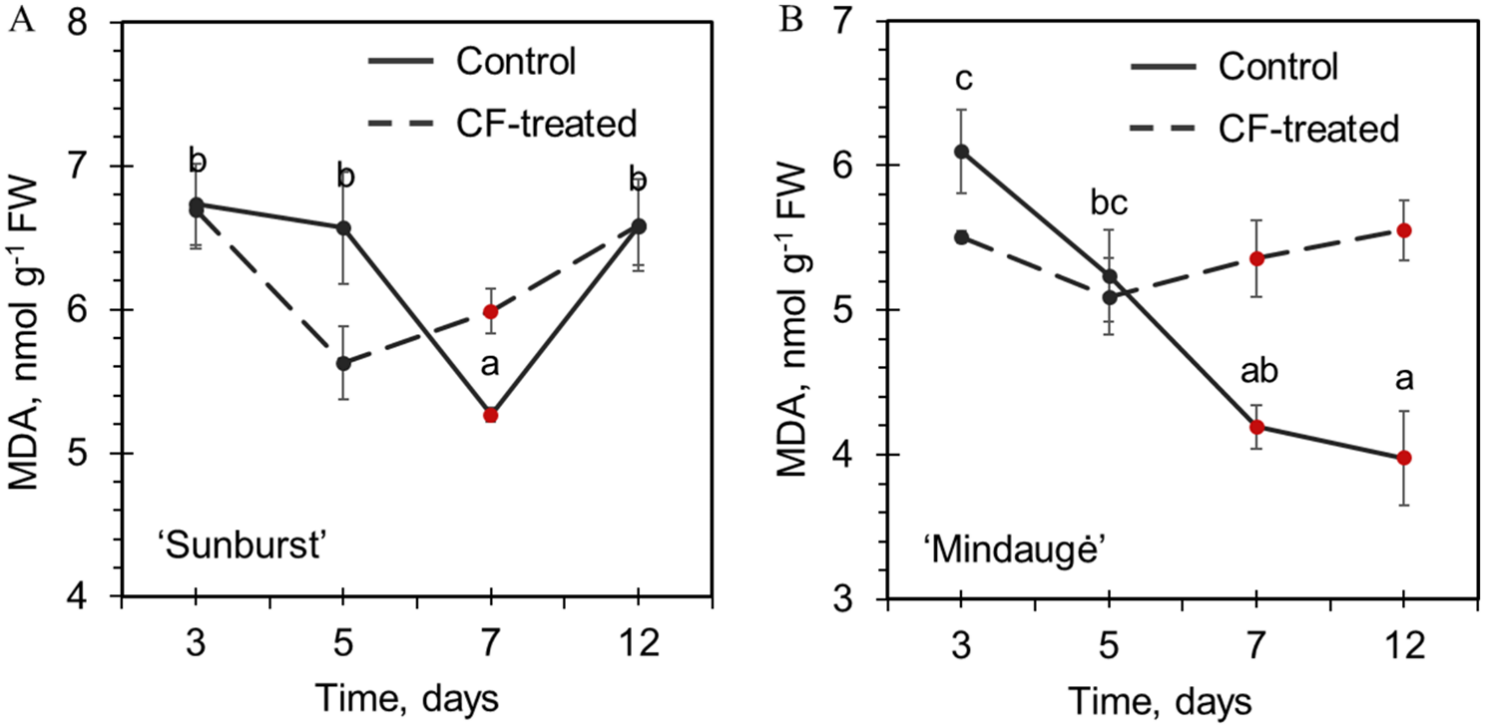
Cryogenic freezing effect on in vitro shoot stress injury level. Malondialdehyde accumulation in control and CF-treated apical shoot tips of sweet cherry cvs. Sunburst (**A**) and Mindaugė (**B**). The time scale is presented as days after shoot transfer to fresh medium; data are presented as the mean ± SEM; dots marked in red represent significant differences between experimental groups at certain time points (*p* ≤ 0.05); the same letter denotes no significant difference among the time points (*p* ≤ 0.05). FW: fresh weight; MDA: malondialdehyde

An analysis of the tissue stress injury of control and CF-treated shoots revealed significant variation in the MDA concentration during the 12-day cultivation period (Fig. 3), which followed a pattern previously reported for a variety of plant in vitro shoot cultures [45, 60, 61]. The highest values (5.5–6.7 nmol g^− 1^ FW) were detected during the first day after transfer to fresh medium, which was likely a consequence of the combined effect of the stress resulting from tissue senescence and injury during transfer to fresh medium. A gradual decrease in the MDA concentration was observed during adaptation and active growth during the first week of cultivation. Subsequently an increase in oxidative lipid injury associated to culture senescence was observed starting from day 5 for CF-treated samples of both cultivars or day 7 for control cv. Sunburst. Notably, cv. Mindaugė had overall lower levels of MDA accumulation, and the control shoots did not exhibit symptoms of tissue senescence during the analysis period. However, the CF-treated shoots of cv. Mindaugė exhibited an increase in MDA and a decrease in adaptive capacity after 5 days. A less pronounced effect of CF on the MDA accumulation pattern was observed for cv. Sunburst.

### Changes in the endophytic bacterial community of in vitro-cultivated and CF-treated shoot tissues

**Fig. 4:**
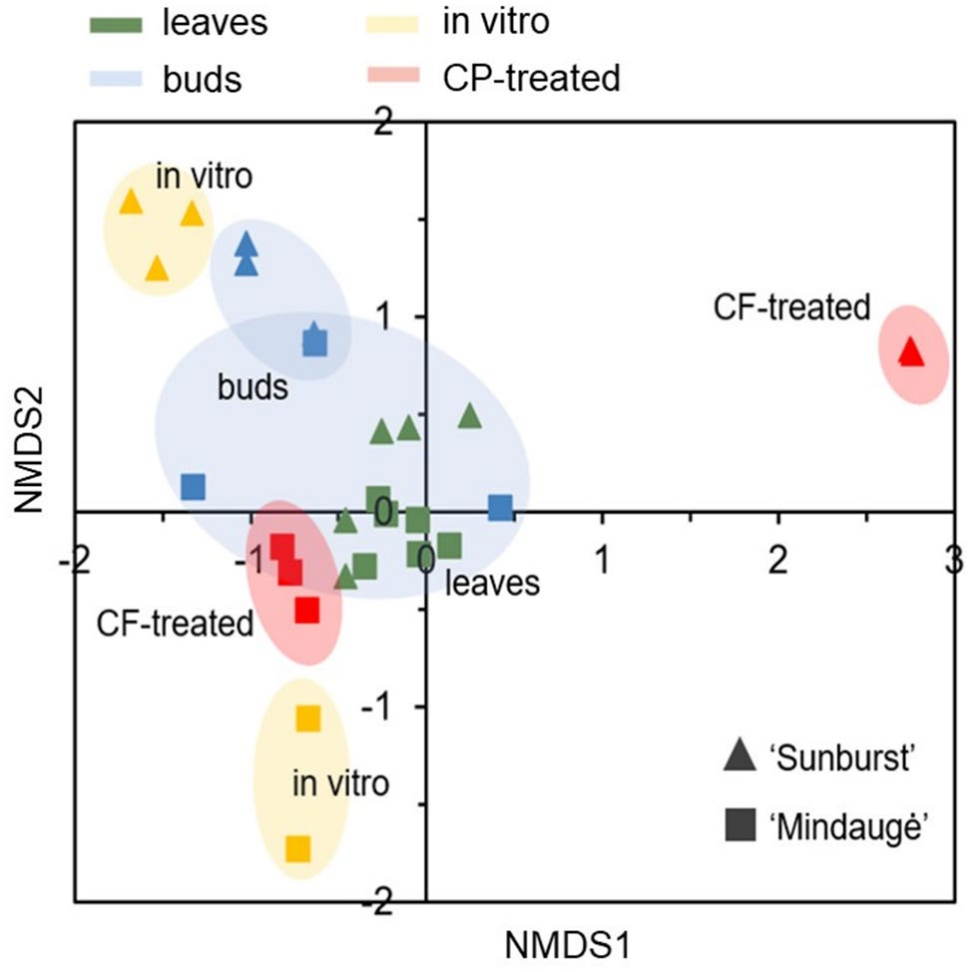
Bacterial diversity variation in sweet cherry leaf, bud, in vitro control, and CF-treated shoot samples. The nonmetric multidimensional scaling (NMDS) analysis of the OTU datasets generated using metataxonomic sequencing of 16 S rRNA variable region V4 of cvs. Sunburst and Mindaugė samples was carried out using the Bray‒Curtis dissimilarity matrix

Endophytic bacteria colonise internal plant tissues and are generally considered to play a crucial role in the stress adaptation of the host plant [62]. Previous studies demonstrated that the reduced abundance and diversity of endophytic bacteria in plant shoots or tissues cultivated under in vitro conditions could also have a negative impact on the growth [63, 64] and adaptation [44, 45] of in vitro plant tissues. In the present study, the endophytic bacterial community composition of in vitro-cultivated sweet cherry shoots and the effect of CF treatment on endophyte diversity were assessed using 16 S rRNA V4 domain sequence-based metataxonomic analysis. Additionally, leaf and bud samples collected from field-grown sweet cherry trees, which were used as the source for in vitro shoot culture initiation, were included in the analyses to assess the impact of the in vitro techniques on the plant microbiome. The 13,664 to 667,706 reads obtained from DNA sequencing were assigned to 1–18 bacterial operational taxonomic units (OTUs) per sample (electronic supplementary material, Table S3, S4). NMDS analysis revealed that leaf samples clustered in close proximity for both, the cvs. Sunburst and Mindaugė, genotypes (Fig. 4). Bud tissue samples had a similar distribution as leaf samples, but more pronounced variation was detected among the samples collected from cv. Mindaugė. Variation increased among in vitro and CF-treated samples compared to samples of leaves and buds collected from field-grown trees. The greatest variation was detected among the in vitro cultivated control and CF-treated samples of cv. Sunburst, while for cv. Mindaugė, the variation among the in vitro control and CF-treated samples was more prominent only on the second axis of NMDS. The results confirmed that in vitro cultivation conditions and CF treatment had a genotype-specific effect on the diversity of the endophytic microbiome.

The abundance of endophytic bacteria in the tissue samples was estimated based on the relative proportion of mapped bacterial reads to plastid and mitochondrial reads. The V4 domain-specific primers used in the analysis show greater specificity for bacterial 16 S rRNA gene sequences than for plant plastid or mitochondrial 16 S rRNA gene sequences; however, the latter are amplified in significant abundance when plastid and mitochondrial DNA are present in large excess compared to bacterial DNA. Although chloroplast and mitochondrial abundance varies among different plant tissues, the ratio of the bacterial 16 S rRNA reads to plastid and mitochondrial reads could provide a rough estimate of bacterial abundance in the sample. Among the sweet cherry samples, the proportion of reads mapped to bacterial OTUs varied 10-fold from 0.6 ± 0.1% to 67.1 ± 5.6% compared to the overall number of mapped reads (including plastids and mitochondria) (Table 1). A low proportion of bacterial reads in the leaf samples of both cultivars could indicate a low abundance of bacteria. This could be expected for the young, emerging leaves used in the analysis. In contrast, in vitro shoot culture had the greatest relative abundance of bacterial DNA, which was similar for both cultivars. Moreover, for the bud and CF-treated shoot samples of cv. Sunburst, the bacterial abundance was estimated to be approximately 80- and 120-fold greater than that of cv. Mindaugė.

**Table 1:** Abundance of bacterial OTUs in sweet cherry samples

Sample	Genotype	
	‘Sunburst’	‘Mindaugė’
Leaves	1.4 ± 0.7	2.2 ± 0.8
Dormant buds	49.7 ± 5.02	0.4 ± 0.01
In vitro shoots	67.1 ± 9.6	52.0 ± 13.5
CF-treated shoots	46.8 ± 7.9	0.6 ± 0.1

A proportion (%) of reads mapped to bacterial OTUs compared to all mapped reads that include plastid and mitochondrial OTUs obtained using 16 S rRNA variable region V4 amplicon sequencing. The data are presented as the mean and standard deviation of three replicates

**Fig. 5:**
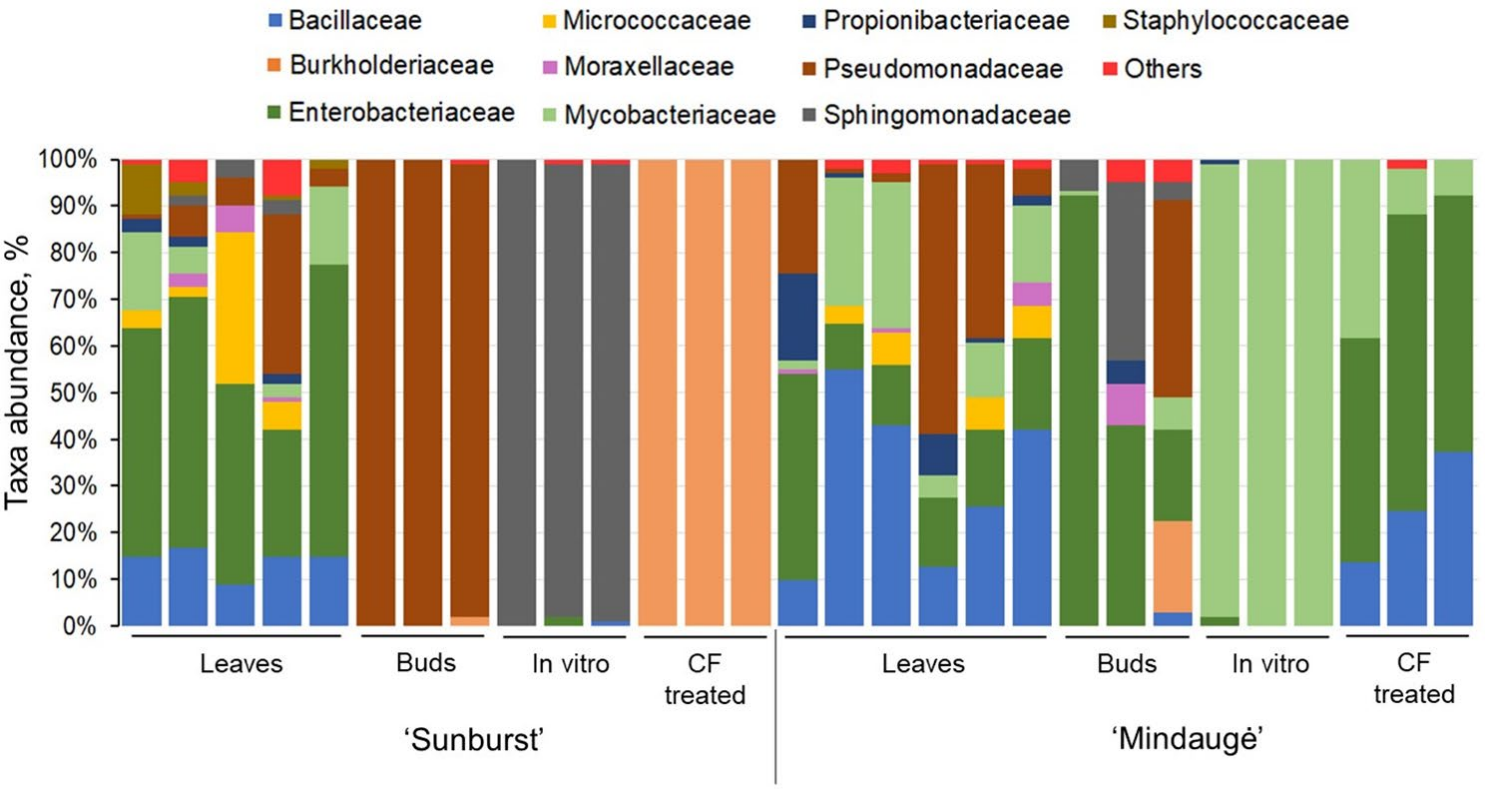
Abundance of endophytic bacterial taxa in leaf, dormant bud, in vitro shoot control, and cryogenic freezing-treated sweet cherry samples. Endophytic bacteria whose abundance did not exceed the 5% level were assigned to others

Further insights into changes induced by in vitro culture and CF treatment were provided by analysis of the diversity and taxonomic composition of the endophytic community. Overall, the bacterial reads were mapped to 35 families, including 21 family detected in at least two experimental groups (Fig. 5; electronic supplementary material, Table S2). The unique families had a low read abundance and were mainly detected in leaf samples (10 families). Two families, *Bacillaceae* and *Enterobacteriaceae*, with variable abundances were detected in all the experimental groups. In addition, *Sphingomonadaceae*, *Pseudomonadaceae*, and *Mycobacteriaceae* were among the most common and, in some cases, dominant taxa. The leaf experimental groups of both genotypes, as well as the bud experimental group of cv. Mindaugė, exhibited a variation in bacterial taxa and their relative abundance among the biological replicates (Fig. 5).

Interestingly, for cv. Sunburst, different dominant taxa were detected for seemingly related experimental groups, e.g., buds and in vitro shoots derived from the dormant buds had *Sphingomonadaceae* (~98%) and *Pseudomonadaceae* (~98%) as predominant taxa, respectively, while *Burkholderiaceae* (100%) prevailed in the shoots after the CF treatment. Among the experimental groups, cv. Mindaugė had more consistent occurrence of the dominant taxa, such as *Bacillaceae* and *Enterobacteriaceae* which were common in the leaves, buds, and shoots after CF treatment, except for in vitro shoots, where *Mycobacteriaceae* became predominant (~99%).

### Effect of pure bacterial isolate inoculation on sweet cherry shoot growth

**Fig. 6:**
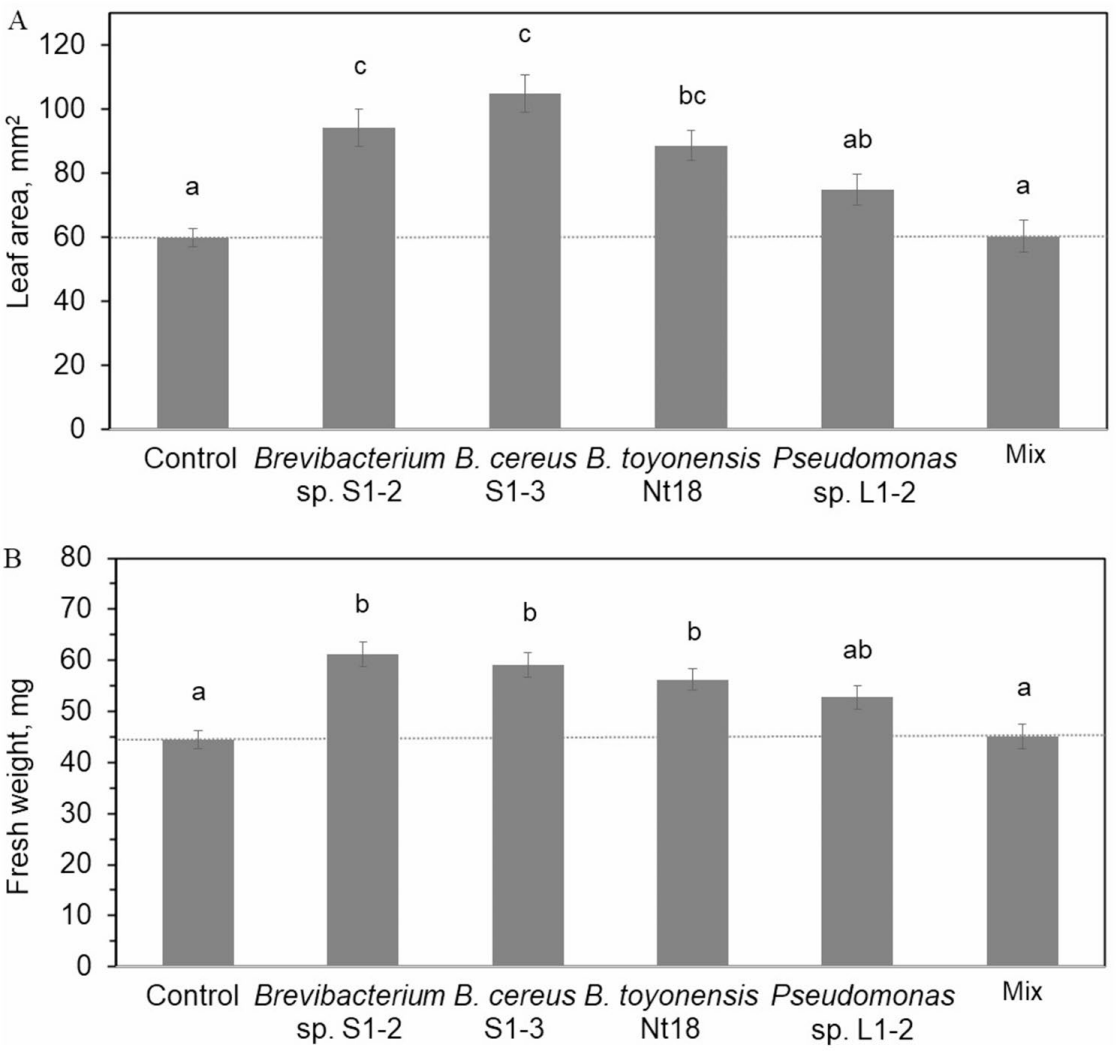
Bacterial isolate cocultivation effect on in vitro shoot growth. Cryogenic freezing-treated sweet cherry cv. Mindaugė in vitro shoots cocultivated with pure bacterial isolates or a mixture of the four isolates and shoot leaf area (**A**) and accumulation of biomass (**B**) measured after 12 days. The data are presented as the mean ± SEM; different letters denote significant differences between experimental groups (*p* ≤ 0.05); a percentage increase compared to the control is indicated for significant differences

To evaluate the potential of plant growth-promoting bacterial isolates to compensate for the reduced performance of in vitro shoots after cryopreservation, cv. Mindaugė shoots were inoculated with isolates of *Brevibacterium* sp. S1-2, *Bacillus cereus* S1-3, *B. toyonensis* Nt18, *Pseudomonas* sp. L1-2, or a mixture of all four isolates. Shoot fresh weight and leaf area were assessed after 12 days of cultivation (Fig. 6). Cocultivation with isolates of *Brevibacterium* and *Bacillus* sp. resulted in a 26% to 37% increase in shoot biomass accumulation and a 48–75% increase in leaf area. Inoculation with *Pseudomanas* sp. isolate had no significant effect on shoot growth. Interestingly, inoculation with a mixture of the four bacterial isolates did not result in a cumulative effect but rather resulted in no significant effect. As none of the bacterial strains included in the mixture had a negative effect on shoot growth, the absence of a growth-promoting effect could be due to antagonistic interactions between the bacterial strains.

## Discussion

Previously, a large variation (11–78%) in recovery rates was reported for distinct *Prunus avium* genotypes after cryopreservation using vitrification with a PVS2 solution [11, 18]. In the present study, similar 10% and 38% regrowth rates were observed for the cvs. Mindaugė and Sunburst, respectively. The difference between the two genotypes was related to the distinct response to shoot preconditioning by cold hardening (Fig. 1; electronic supplementary material, Table S1). Although both genotypes are considered winter hardy and perform well under northern temperate climate conditions [65], cold acclimation treatment improved the survival and regrowth of apical shoot tips only of cv. Sunburst and did not affect the shoots of cv. Mindaugė. This is likely a consequence of different cold acclimation traits related to differences in the genetic backgrounds of the two genotypes. The two genotypes used in our study have a partially established pedigree that includes interrelated traditional European (e.g., French, British, and German) cultivars (electronic supplementary material, Fig. S9) [66, 67]; however, the most notable difference is that one of the ancestors of cv. Mindaugė includes cv. Severnaya, whose origin is described as an open pollination seedling. Previous analysis of sweet cherry genetic diversity showed that cv. Severnaya clusters together with the landraces Žemaičių rožinė and Žemaičių geltonoji [68], which are common in the region where cv. Severnaya was developed. These cherries have similar yellow and pink fruits; however, none of the detected rare microsatellite alleles characteristic of the landraces were present in Severnaya [68], casting uncertainty about the direct relationship between the genotypes. Therefore, further assessment of the pedigree and/or analysis of genetic mechanisms involved in the cold hardening response of the two cultivars is required to explain the genetic basis responsible for the cold hardening-related differences resulting in different regrowth after cryopreservation.

Plant species- or genotype-specific endophytic microbial communities are essential for maintaining plant health and productivity; therefore, in addition to plant germplasm cryopreservation, the preservation of endophytic biodiversity might present a useful approach to improve the effective recovery and performance of plant germplasm after conservation, especially from a long-term perspective. Groundbreaking work on the conservation of plant-associated microbiota diversity has been carried out by the UK Crop Microbiome Cryobank initiative, which is focused on the cryopreservation of soil microbial communities associated with major crops [69]; however, such an approach does not include unculturable endophytic microbiota. To date, cryopreservation of host plant tissues has been successfully applied to plant viruses and viroids that cannot be maintained using cultivation techniques [70, 71]. To determine whether the preservation of endophytic bacteria in host tissue is feasible, in the present study, we assessed the effect of in vitro cultivation and cryopreservation procedures on the composition of the endophytic bacterial community. Metataxonomic analysis revealed a variation in bacterial abundance and diversity between the two genotypes as well as among the samples collected from field and in vitro shoot culture before and after CF treatment (Table 1; Fig. 5). *Bacillaceae* and *Enterobacteriaceae* were abundant in leaf samples of both genotypes and dormant buds of cv. Mindaugė. Although there is limited information about the endophytic microbiome of field-grown *Prunus* sp. trees, *Bacillacea* and *Enterobacteriaceae* were previously described as the dominant microbiota on sweet cherry fruit surfaces [72]. A variation in *Enterobacteriaceae* abundance from detectable to predominant was also described for individual almond trees [73]. The variation in the low abundance of bacteria detected in leaf samples could be a result of differences in endophyte colonisation among the individual trees used in the study, which was previously described for the *Prunus* leaf microbiome [73]. Additionally, it could also be at least partially an artefact of sample contamination with a relic DNA of bacteria residing in the leaf phylloplane that could not be removed by surface washing and sterilisation procedures.

Interestingly, *Pseudomonadaceae* was detected as the predominant family in the buds of cv. Sunburst, which could indicate infection by bacterial canker-inflicting strains of *Pseudomonas syringae.* This pathogen can colonise sweet cherry and other stone fruit through leaf scar infection and subsequently migrate to dormant buds [74, 75]. However, due to the limited taxonomic depth of short amplicon sequencing, species or strains of the detected *Pseudomonadaceae* could not be identified.

As expected, crucial changes in bacterial composition and abundance were observed between the leaves or buds of field-grown trees and in vitro-grown tissues. Although sweet cherry in vitro shoot culture was initiated from dormant buds of field-grown trees, its microbial composition barely resembled the source, and *Sphingomonadaceae* and *Mycobacteriaceae* were found to largely dominate in in vitro shoot culture of cvs. Sunburst and Mindaugė, respectively. Loss of microbial diversity in vitro could be partially related to disruption of the endophytic microbiome composition by the sterilisation procedure used for in vitro culture initiation as well as by unfavourable in vitro conditions, such as acidic medium, a lack of nutrients, or changes in host plant physiology. This could be overcome by endospore- or biofilm-forming species of bacteria that are adapted to survive in unfavourable environments. In the case of sweet cherry in vitro shoot culture, such a route of colonisation could explain the dominance of the *Sphingomonadaceae* and *Mycobacteriaceae* families for distinct genotypes. Members of the *Sphingomonadaceae* family are high polysaccharide producers and typical biofilm-forming bacteria [76]; meanwhile, species of the *Mycobacteriaceae* family include a variety of common soil and water that form resilient endospores that are resistant to sterilising agents [77]. Notably, *Mycobacterium* have also been described as endophytic species in a variety of plants [78–84]. Conversely, bacteria of the *Mycobacterium* genus have been described as widespread contaminants in plant tissue cultures of ornamentals [85], sweet cherry [64], tobacco shoot culture [45], and Scots pine tissue cultures [86]. According to Quambush et al. [64], *Mycobacterium* sp. was the most prominent bacterial genus found in tissue culture of several *P. avium* genotypes and it was predominant in the difficult-to-propagate genotypes, suggesting that these bacteria could have a detrimental effect on in vitro cultures of *Prunus*.

Subsequent radical changes in the sweet cherry shoot endophytic bacterial composition were induced by CF treatment. *Burkholderiaceae* became highly prevalent in the shoots of cv. Sunburst. Interestingly, for cv. Mindaugė, *Mycobacteriaceae* was replaced by the *Bacillaceae* and *Enterobacteriaceae* families, and the composition of the CF-treated shoots became partially similar to that of the leaf samples. The *Burkholderiaceae* family includes a variety of bacteria widely distributed in the environment, among which more than thirty nonpathogenic species have been shown to live in close association with plants, including endophytic colonisers [87, 88]. Moreover, the dominance of *Sphingomonadaceae* and *Mycobacteriaceae* in sweet cherry in in vitro shoot culture could be rationalised by the capacity of representatives of these families to propagate under conditions unfavourable for bacterial growth, as circumstances that lead to the dominance of *Burkholderiaceae* in CF-treated shoots are less obvious. It could be presumed that such variation in dominant microbial species between distinct genotypes or in vitro cultures could be a random phenomenon that is to some extent dependent on variation in plant physiology, e.g., the in vitro stress-induced dysbiosis effect, which leads to the inability of plants to regulate interactions with microorganisms and the dominance of certain microbial species. This finding was consistent with the reduced growth rate and elevated levels of oxidative stress injury detected in the CF-treated cultures of both genotypes (Figs. 2 and 3). However, it is noteworthy that improved grapevine tolerance to cold and postchilling recovery by the colonisation of plants with endophytic *Burkholderia phytofirmans* strain PsJN was previously described [89–91]. Therefore, further investigation is required to address the potential relationship between *Burkholderiaceae* bacterial colonisation of shoot culture of cv. Sunburst and increased tolerance to CF treatment.

Microorganisms play an important role in plant adaptation and growth regulation. In recent years, the use of beneficial microbes to enhance plant growth and development has been the subject of many studies, including applications for in vitro culture [92]. Previous research on a variety of plants [43, 46], including sweet cherry [64], revealed that plant-microbial interactions could be at least partially maintained under aseptic in vitro conditions and could have a beneficial effect on the performance of in vitro shoot or tissue culture. In addition, the application of plant growth-promoting bacterial inocula for plant micropropagation, rooting in vitro, and acclimation ex vitro has been reported (overviewed in Orlikowska et al. [37], Soumare et al. [93] and Cantabella et al. [92]). *Bacillus* sp. bacteria have been shown to promote transgenic tobacco shoot culture growth in vitro [44] and the growth of banana [94], strawberry [95], and grapevine [96] plants. In our study, cocultivation of *Brevibacterium* sp. and *Bacillus* sp. isolates with CF-treated shoots of cv. Mindaugė mitigated the growth-suppressing effect of cryopreservation treatment and restored the shoot culture growth rate to the levels observed for the untreated control shoots. The results demonstrate the potential application of pure endophytic bacterial isolates as inoculum to improve the recovery and adaptation of in vitro cultures after cryopreservation.

## Conclusions

In conclusion, this research presents novel insights into the variation in the diversity and abundance of endophytic bacteria between aerial organs of field-grown sweet cherry tree plants and in vitro propagated and CF-treated shoot tissues. This study revealed that endophytic bacterial diversity is significantly reduced under in vitro conditions, often leading to a genotype-specific increase in the abundance and dominance of bacteria attributed to a single bacterial family. Our findings suggest that in vitro cultivation and cryopreservation-induced changes in endophyte composition could be related to the variation in in vitro shoot physiology induced by factors such as genotype-specific responses to cold-hardening preconditioning or CF procedures and manifested as increased stress injury and suppression of growth. The results emphasise the role of the complex interplay between plant physiology and environmental factors in shaping interactions with the endophytic microbial community. Moreover, shoot cocultivation experiments with pure endophytic bacterial isolates demonstrated the potential application of plant growth-promoting bacteria inoculum to improve the recovery of plant shoots after cryopreservation. These findings open up new possibilities for improved propagation and cryopreservation of clonally propagated horticultural plants using in vitro and CF techniques.

## Electronic supplementary material

Below is the link to the electronic supplementary material.

Supplementary Material 1

Supplementary Material 2

## Data Availability

Sequence data that support the findings of this study have been deposited in the NCBI Sequence Read Archive with the primary accession code PRJNA1139108, https://www.ncbi.nlm.nih.gov/sra/PRJNA1139108.

## Abbreviations

CF: Cryogenic Freezing
CFUs: Colony-Forming Units
CP: Cryoprotector
FW: Fresh Weight
LB: Lysogeny Broth
MDA: Malondialdehyde
MS: Murashige and Skoog
NMDS: Nonmetric Multidimensional Scaling
OTUs: Operational Taxonomic Units
PVS2: Plant Vitrification Solution
SEM: Standard Error of the Mean
