# Supplementary material for: Endophytic bacterial community dynamics in sweet cherry in vitro shoot culture and their role in shoot adaptation after cryopreservation
Source: BMC Plant Biol. 2024 Nov 29;24:1145. doi: 10.1186/s12870-024-05866-z (PMC11606084; doi:10.1186/s12870-024-05866-z)
Supplement: Supplementary file 2 — Supplementary Material 2 [file 12870_2024_5866_MOESM2_ESM.docx]

**Supplementary Materials**

**Endophytic bacterial community dynamics in sweet cherry *in vitro* shoot culture and their role in shoot adaptation after cryopreservation**

**Jurgita Vinskienė, Inga Tamošiūnė, Rytis Rugienius, Elena Andriūnaitė, Vidmantas Stanys and Danas Baniulis***

**Institute of Horticulture, Lithuanian Research Centre for Agriculture and Forestry, Kaunas str. 30, Babtai, 54333 Kaunas distr., Lithuania**

**Tables**

**Table S1.** **The survival and regrowth rates of cryoprotector pretreated (CP) and cryogenic freezing-treated (CF) apical shoot tips of sweet cherry cvs. Sunburst and Mindaugė with or without pre-conditioning treatment.**

| **Survival** | | | | | | |
| --- | --- | --- | --- | --- | --- | --- |
| Preconditioning | Unhardened | | | Hardened | | |
| CP/CF | +/- | +/+ | | +/- | +/+ | |
| ‘Sunburst’ | 28.7±23.6 (100) | 3.9±4.5 (110) | | 70.9±16.3 (160) | 69.3±18.9 (130) | |
| ‘Mindaugė’ | 32.2±11.5 (90) | 42.9±11.1 (70) | | 74.4±18.1 (90) | 45.9±18.1 (100) | |
| **Regrowth** | | | | | | |
| Preconditioning | Unhardened | | | Hardened | | |
| CP/CF | +/- | | +/+ | +/- | | +/+ |
| ‘Sunburst’ | 18.6±19.1 (40) | | 4.3±4.6 (100) | 42.6±21.6 (150) | | 42.5±23.1 (140) |
| ‘Mindaugė’ | 10.0±8.2 (40) | | 19.3±8.2 (80) | 51.7±27.1 (60) | | 11.9±14.3 (80) |

Preconditioning treatment: *in vitro* shoots were cold hardened at 4 °C for two weeks before the preparation of apical shoot tips. Unhardened shoots were grown under *in vitro* shoot propagation conditions as described in the Materials and methods section. Cryoprotector pretreatment (CP) conditions: apical shoot tips after excision were incubated on preculture MS medium at 22 °C for two days. CP-treated apical shoot tips were exposed to cryoprotectant treatment, but not cryogenic freezing and thawing steps; cryogenic freezing-treated (CF) apical shoot tips were exposed to cryoprotectants, cryogenic freezing, and thawing steps. The data is presented as the mean and standard deviation; the number of apical shoot tips used in the experiment is indicated in the brackets. Survival was estimated after one month of incubation and regrowth was assessed after one month of cultivation.

**Table S2. Alpha diversity indices of different sweet cherry cvs. Sunburst and Mindaugė samples using 16S rRNA sequencing analysis.**

|  | ‘Sunburst’ | | | | ‘Mindaugė’ | | | |
| --- | --- | --- | --- | --- | --- | --- | --- | --- |
| Diversity index | Leaves | Buds | *In vitro* | CF-treated | Leaves | Buds | *In vitro* | CF-treated |
| Shannon | 1.49±0.21^a^ (5) | 0.13±0.18^b^ (3) | 0.04±0.05^b^ (3) | 0.03±0.00^b^ (3) | 1.40±0.16^a^ (6) | 1.04±0.65^a^ (3) | 0.05±0.09^b^ (3) | 0.94±0.05^a^ (3) |
| p-value: 0.00000017; F-value: 88.562 | | | | | p-value: 0.00053; F-value: 13.443 | | | |
| Simpson | 0.67±0.07^a^ (5) | 0.05±0.07^b^ (3) | 0.01±0.02^b^ (3) | 0.01±0.00^b^ (3) | 0.69±0.06^a^ (6) | 0.51±0.32^a^ (3) | 0.02±0.03^b^ (3) | 0.56±0.04^a^ (3) |
| p-value: 0.000000019; F-value: 139.34 | | | | | p-value: 0.00037; F-value: 14.666 | | | |

Statistical data analysis was performed using the Microbiome Analyst server (Chong et al., 2020). Data is presented as the mean and standard deviation; the number of biological replicates used in the experiment is indicated in the brackets. Different letters indicate significant differences between analysed groups according to one-way ANOVA Tukey’s multiple comparison test (*p* ≤ 0.05).

**Figures**


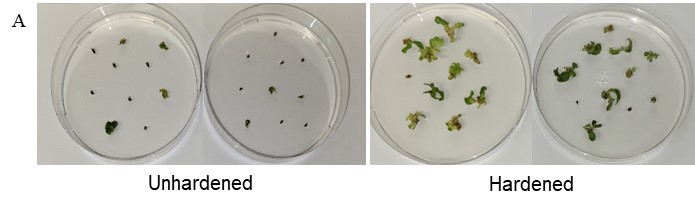

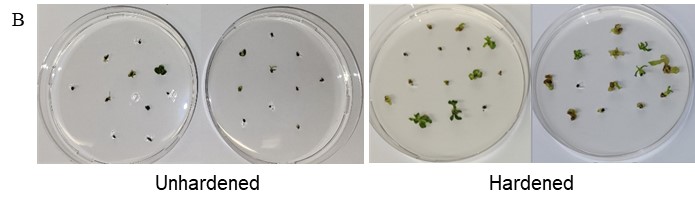


**Figure S1. Regrowth of sweet cherry cv. Sunburst apical shoot tips after cryoprotector pretreatment (A) and cryogenic freezing-treatment (B) with or without cold hardening preconditioning treatment.**


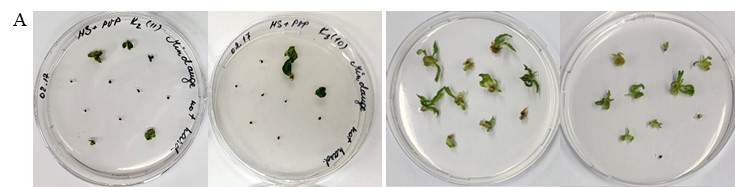

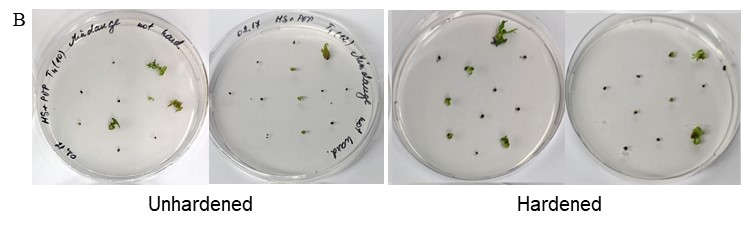


**Figure S2. Regrowth of sweet cherry cv. Mindaugė apical shoot tips after cryoprotector pretreatment (A) and cryogenic freezing-treatment (B) with or without cold hardening preconditioning treatment.**


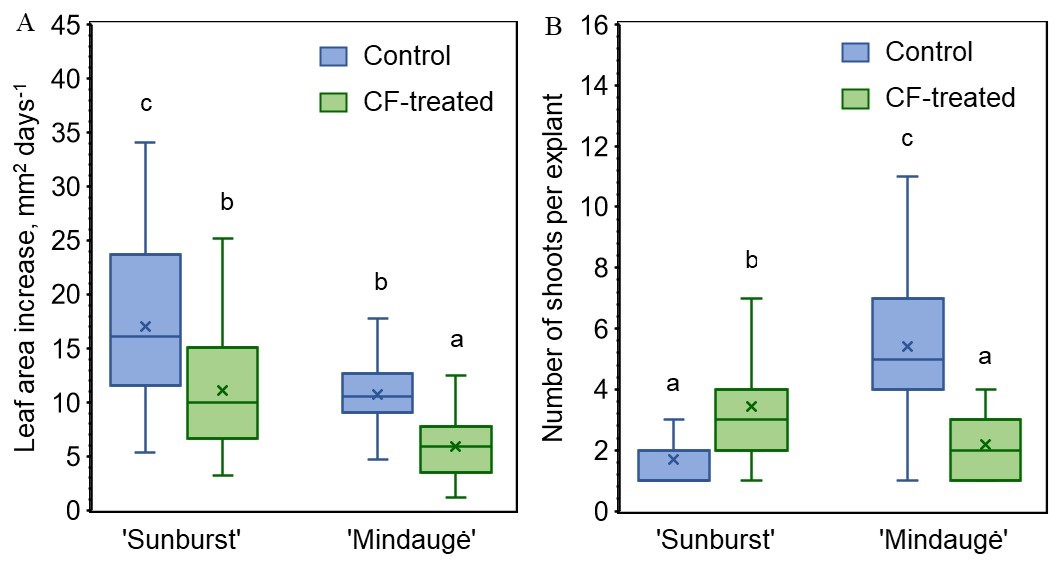


**Figure S3. Cryogenic freezing effect on in vitro shoot leaf area increase rate (A) and shoot proliferation rate (B) of control and shoot regenerated from CF-treated apical shoot tips of sweet cherry cvs. Sunburst and Mindaugė.** The data are shown as boxplots representing the means, medians, minimum and maximum scores, and lower and upper quartiles; different letters denote significant differences between the analysed groups (ANOVA, p ≤ 0.05).


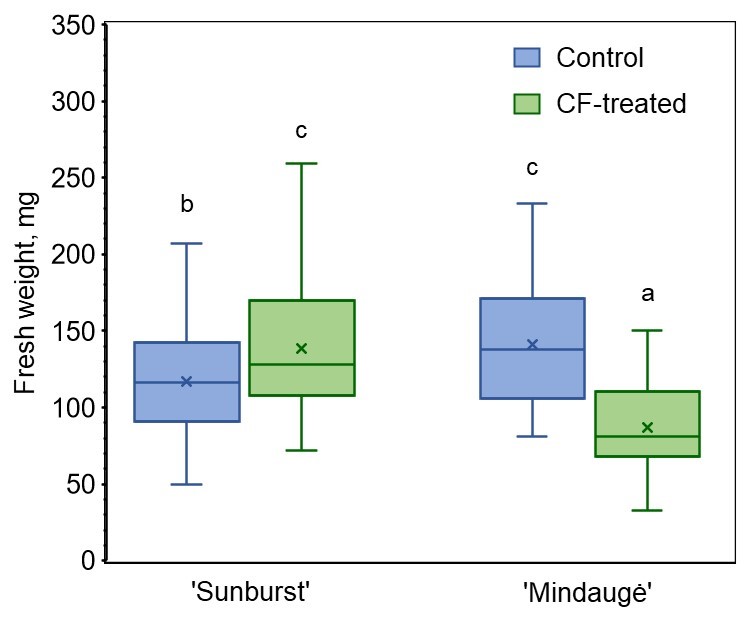


**Figure S4. Fresh weight of shoots grown under *in vitro* shoot propagation conditions (control) and cryogenic freezing-treated apical shoot tips (CF-treated) of sweet cherry cvs. Sunburst and Mindaugė after three weeks of cultivation.**

Different letters indicate significant differences between analysed groups (*p* ≤ 0.05).


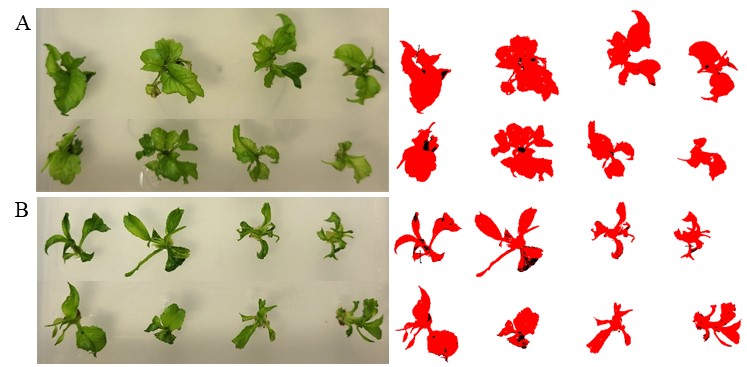

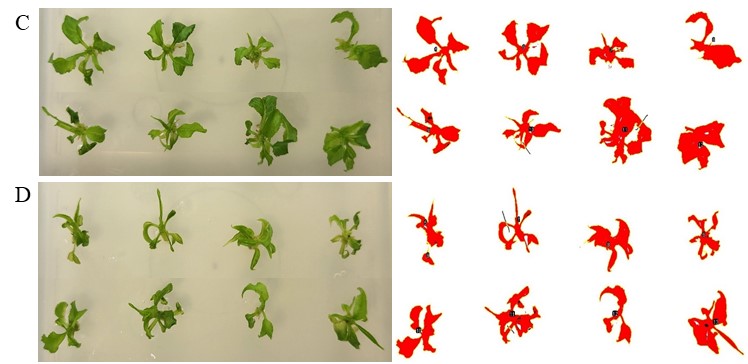


**Figure S5. Sweet cherry cvs. Sunburst (A, B) and Mindaugė (C, D) control shoots grown under *in vitro* shoot propagation conditions (A, C) and cryogenic freezing-treated apical shoot tips (B, D) after three weeks of cultivation (left panel). Leaf area estimate using ImageJ software (Schneider et al., 2012) (right panel).**


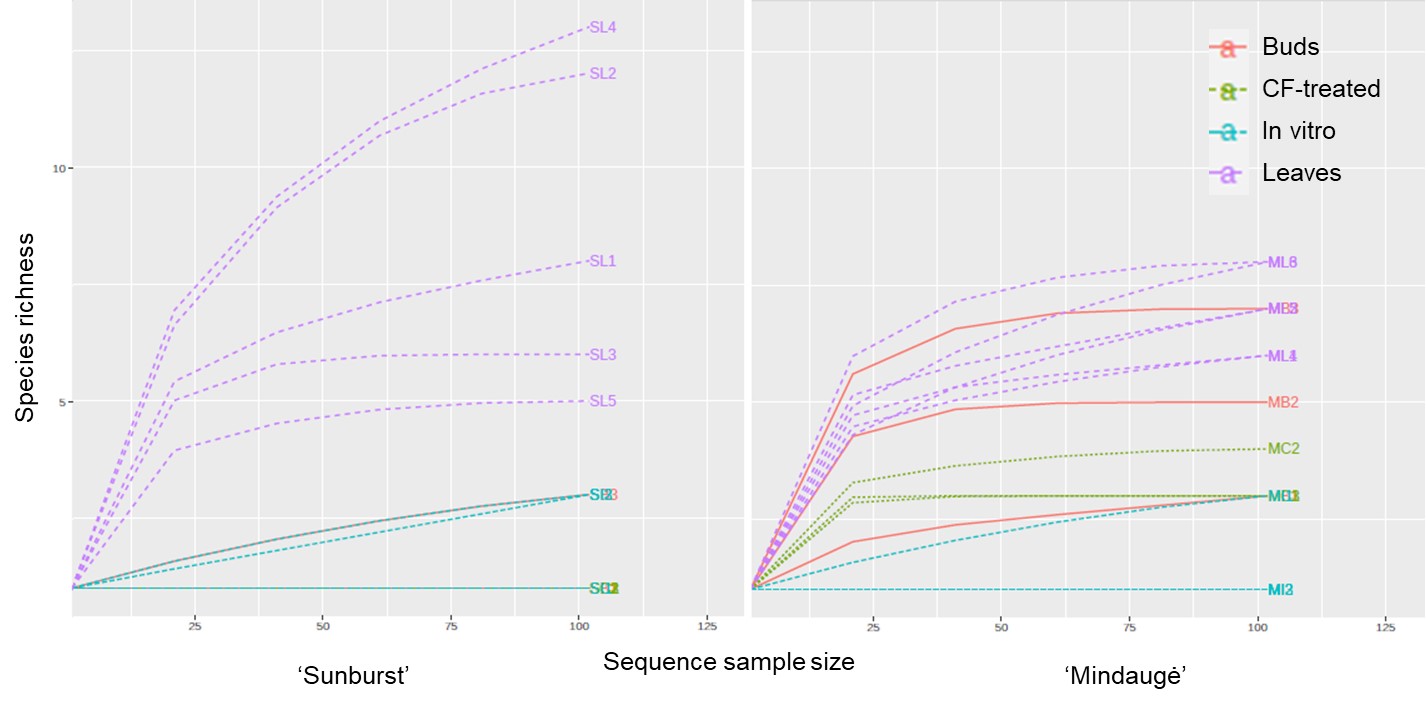


**Figure S6. Rarefaction curves for leaf, dormant bud, in vitro shoot control, and cryogenic freezing-treated samples of sweet cherry cvs. Sunburst and Mindaugė after 16S rRNA high-throughput sequencing analysis estimated using the Microbiome Analyst server (Chong et al., 2020).**


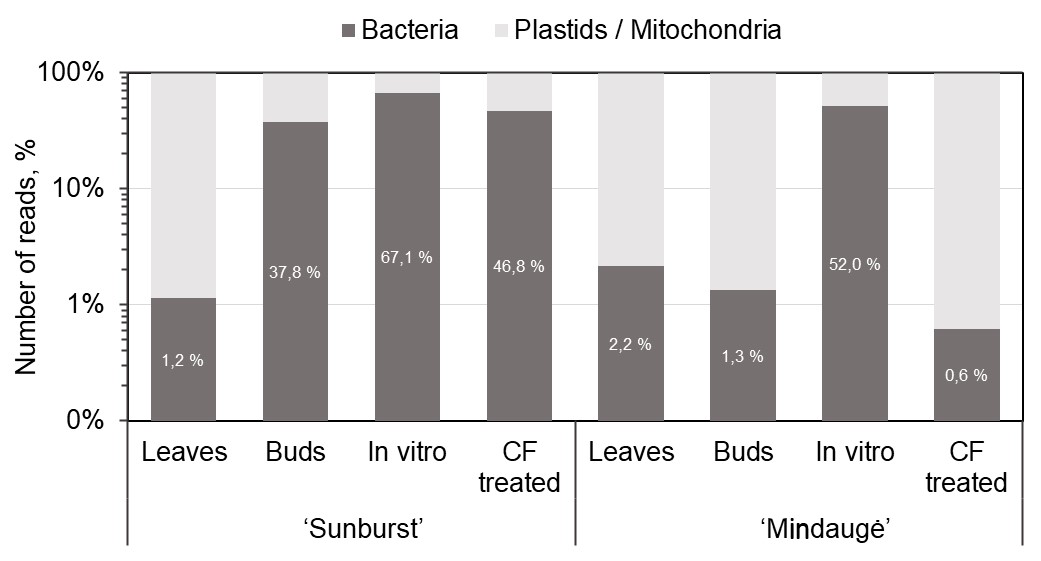


**Figure S7. Distribution of the total number of mapped reads and proportion of bacterial OTUs (dark gray) to plastid or mitochondrial 16S rRNA sequences (light gray) estimated in leaf, dormant bud, in vitro shoot control, and cryogenic freezing-treated samples of sweet cherry cvs. Sunburst and Mindaugė.**


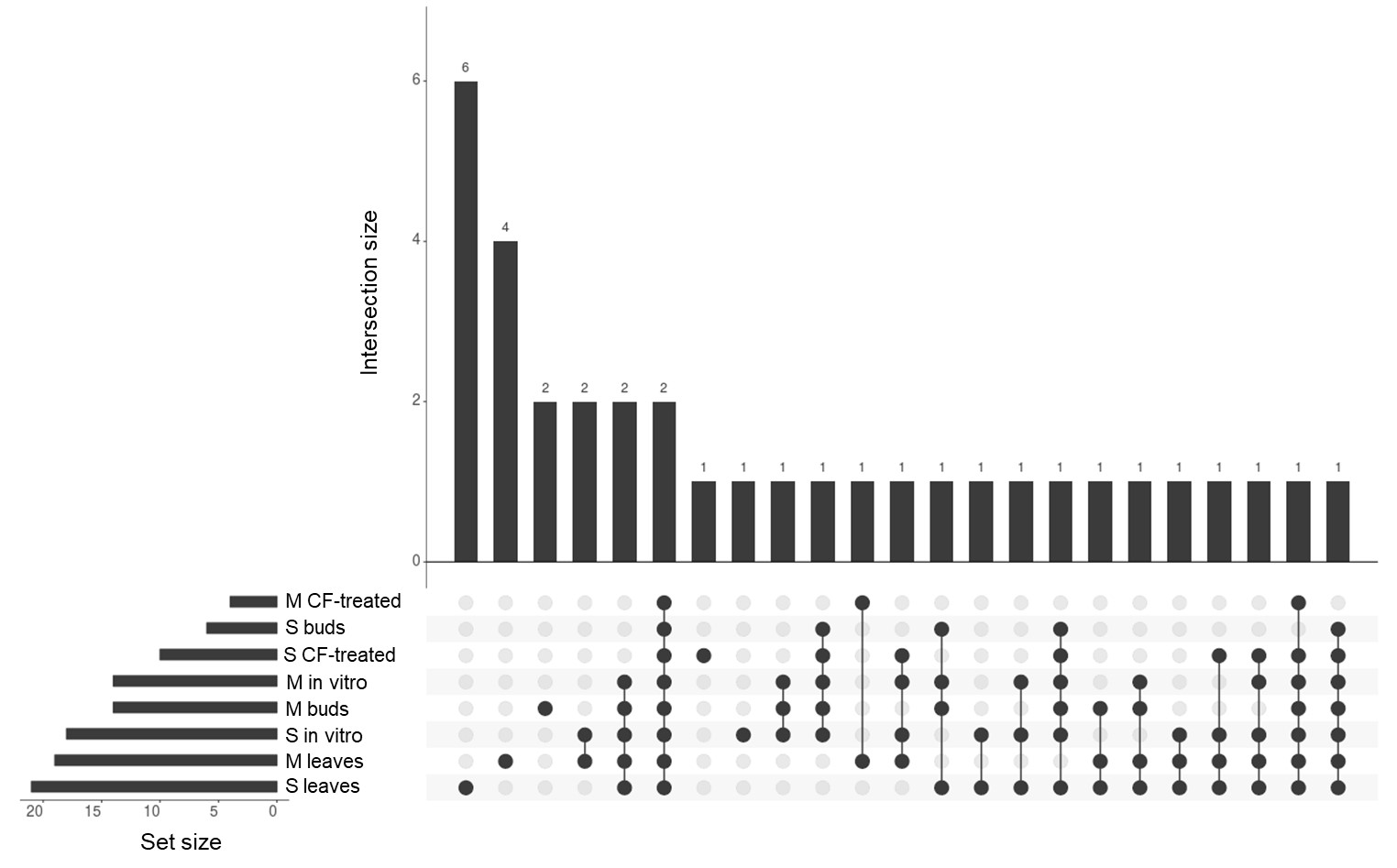


**Figure S8. Association Upset plot representing endophytic bacteria family-level data in leaf, dormant bud, in vitro shoot control, and cryogenic freezing-treated samples of sweet cherry cvs. Sunburst (S) and Mindaugė (M).**


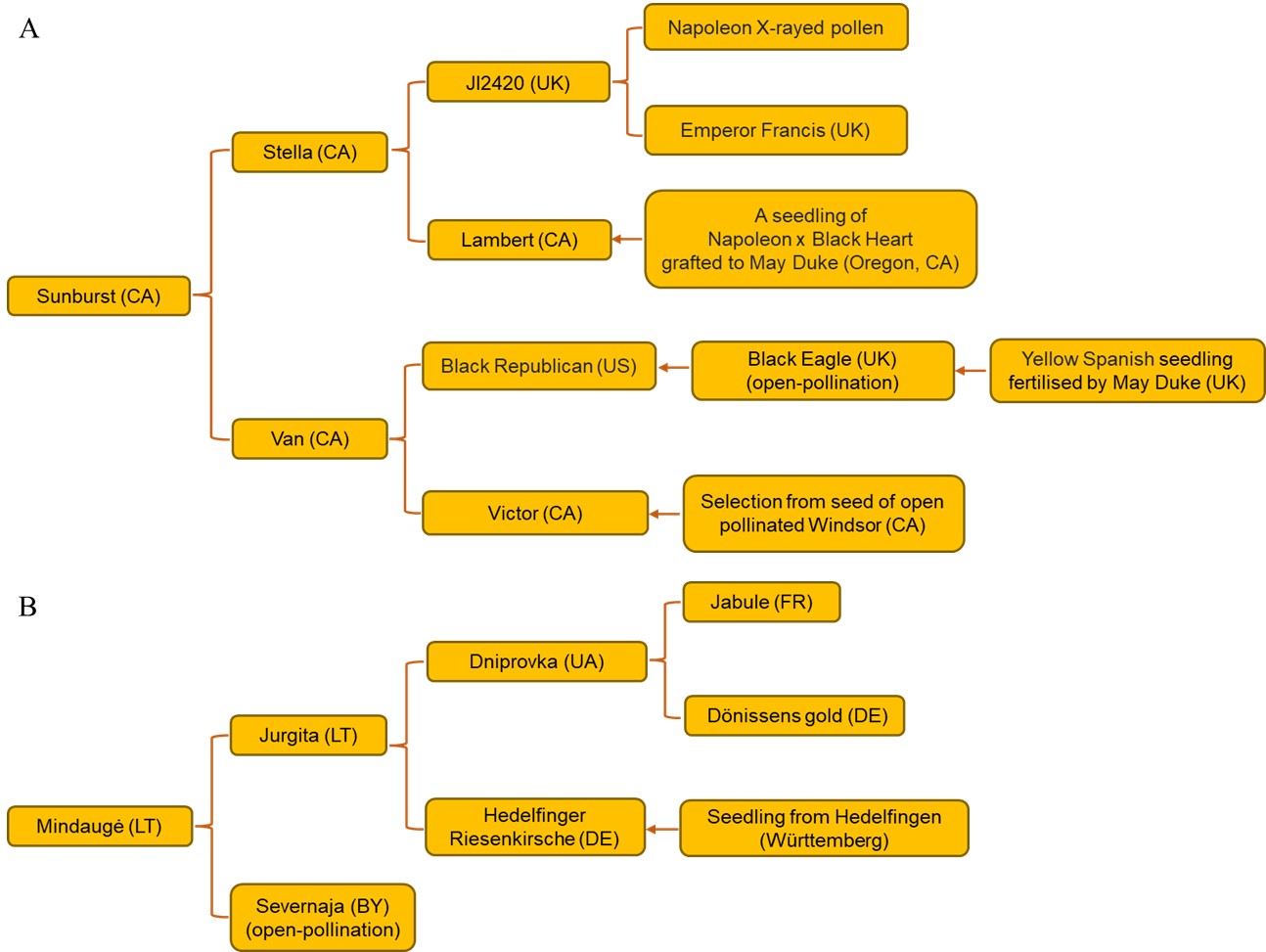


**Figure S9. The genealogy of sweet cherry cvs. Sunburst (A) and Mindaugė (B).**
